# Supplementary material for: Potential of artificial intelligence to accelerate diagnosis and drug discovery for COVID-19
Source: PeerJ. 2021 Oct 5;9:e12073. doi: 10.7717/peerj.12073 (PMC8500072; doi:10.7717/peerj.12073)
Supplement: Supplemental Information 1 [file peerj-09-12073-s001.docx]

**Supplementary Table 1: Search strategy for literature review**

| **Database** | **Search queries** |
| --- | --- |
| **Google scholar** | **On SARS-CoV-2:** COVID-19 symptoms, SARS-CoV-2 infection, SARS-CoV-2 pathology, RT-PCR test for COVID-19, Chest CT for COVID-19, Immunoassay, radiology, ophthalmology  **On Artificial Intelligence:** Machine learning, neural networks, Drug discovery using AI, AI tools.  **On Protein prediction:** Homology modelling, Protein prediction |
| **Nature** | **On SARS-CoV-2:** COVID-19 symptoms, SARS-CoV-2 infection, SARS-CoV-2 pathology, AI tools. |
| **WHO** | **On SARS-CoV-2:** COVID-19 symptoms, SARS-CoV-2 infection |
| **Pubmed** | **On SARS-CoV-2:** COVID-19 symptoms, SARS-CoV-2 pathology, RT-PCR test for COVID-19.  **On Artificial Intelligence:** Machine learning, neural networks, Drug discovery using AI, AI tools. |
